# On the reliability and the limits of inference of amino acid sequence alignments

**DOI:** 10.1093/bioinformatics/btac247

**Published:** 2022-06-27

**Authors:** Sandun Rajapaksa, Dinithi Sumanaweera, Arthur M Lesk, Lloyd Allison, Peter J Stuckey, Maria Garcia de la Banda, David Abramson, Arun S Konagurthu

**Affiliations:** Department of Data Science and Artificial Intelligence, Faculty of Information Technology, Monash University, Clayton, VIC 3800, Australia; Department of Data Science and Artificial Intelligence, Faculty of Information Technology, Monash University, Clayton, VIC 3800, Australia; Department of Biochemistry and Molecular Biology and Center for Computational Biology and Bioinformatics, The Pennsylvania State University, University Park, PA 16802, USA; Department of Data Science and Artificial Intelligence, Faculty of Information Technology, Monash University, Clayton, VIC 3800, Australia; Department of Data Science and Artificial Intelligence, Faculty of Information Technology, Monash University, Clayton, VIC 3800, Australia; Department of Data Science and Artificial Intelligence, Faculty of Information Technology, Monash University, Clayton, VIC 3800, Australia; Research Computing Center, University of Queensland, St Lucia, QLD 4067, Australia; Department of Data Science and Artificial Intelligence, Faculty of Information Technology, Monash University, Clayton, VIC 3800, Australia

## Abstract

**Motivation:**

Alignments are correspondences between sequences. How reliable are alignments of amino acid sequences of proteins, and what inferences about protein relationships can be drawn? Using techniques not previously applied to these questions, by weighting *every* possible sequence alignment by its posterior probability we derive a formal *mathematical expectation*, and develop an efficient algorithm for computation of the distance between alternative alignments allowing quantitative comparisons of sequence-based alignments with corresponding reference structure alignments.

**Results:**

By analyzing the sequences and structures of 1 million protein domain pairs, we report the variation of the expected distance between sequence-based and structure-based alignments, as a function of (Markov time of) sequence divergence. Our results clearly demarcate the ‘daylight’, ‘twilight’ and ‘midnight’ zones for interpreting residue–residue correspondences from sequence information alone.

**Supplementary information:**

Supplementary data are available at *Bioinformatics* online.

## 1 Introduction

Evolution introduces mutations, insertions and deletions at the level of individual protein domains. These changes flow down, in varying degrees, causing perturbations in observed three-dimensional structures. The effect that these perturbations have on the biological functions of proteins influences the fitness and evolutionary selection of these changes. This mechanism of heritable changes is a driver of the Darwinian descent of species through modification (Lesk, 2016; Zuckerkandl and Pauling, 1965).

Comparing related proteins by means of their *alignment* helps scientists understand the cumulative evolutionary events on extant proteins and gain insights into the patterns of conservation and divergences of sequences and their effect on structure and function. Therefore, it is common to see protein alignments as a first step supporting subsequent downstream analyses in biology (Doolittle, 1986).

An alignment is simply a hypothesis of a residue–residue correspondence between protein sequences. The corresponding amino acid residues can imply they likely descended from the same locus in the genome of their common ancestor (Konagurthu *et al.*, 2006). *Sequence-based alignments* use the amino acid sequence information to assert a residue–residue relationship, whereas *structure alignments* use the (*x*, *y*, *z*) atomic coordinates of their corresponding three-dimensional structures.

Inferring reliable residue–residue relationships for proteins using sequence-based alignments is a challenging problem even when the sequences have diverged only moderately in evolution (Do *et al.*, 2006; Doolittle, 1992; Rivas and Eddy, 2015; Sumanaweera *et al.*, 2019). In contrast, relationships derived from structure alignments are significantly more reliable, as structure is far more conserved in evolution than sequence (Konagurthu *et al.*, 2006; Lesk, 2016). The reliability of structure alignments has helped the curation of structural classification databases, such as SCOP (Murzin *et al.*, 1995), where homology between protein domains can be reliably asserted even when their amino acid sequences have changed beyond recognition. However, in the absence of structural information, sequence alignments remain the main port of call to understand evolutionary relationships.

Nevertheless, popular sequence alignment programs have caveats resulting from their underlying assumptions and models of computation. It is thus important for users of these programs to understand that these caveats exist and, as a result, the generated alignments should not be seen as a definitive statement of a relationship between proteins. This is particularly important for programs that generate a *single* alignment as a statement of the proposed residue–residue relationship. The effect of these caveats is reflected in the contradictory nature of the sequence alignments generated by modern programs, as highlighted by several comparative surveys (Do *et al.*, 2006; Rivas and Eddy, 2015; Sumanaweera *et al.*, 2019; Vingron and Waterman, 1994). This problem is exacerbated by the sensitivity of these programs to ‘tunable’ parameters that allow users to select some *fixed* amino acid substitution matrix (used to score the matched amino acid correspondences) and corresponding gap parameters (used to penalize the unmatched amino acids within the sequences). Such parameter-tinkering has significant effects on the reliability of sequence alignments, as clearly demonstrated by comparative surveys (Barton and Sternberg, 1987; Blake and Cohen, 2001; Fitch and Smith, 1983; Sumanaweera *et al.*, 2019; Vingron and Waterman, 1994). All these issues affect the trustworthiness of resultant sequence alignments.

But even if none of these issues occurred, there is a natural limit of inference to deciphering a residue-level alignment relationship based on pairs of protein sequences alone. Doolittle (1986) coined the term *twilight zone* of protein pairwise sequence relationships and defined it as a range of divergence where any single statement of a residue–residue correspondence becomes tenuous and highly untrustworthy (Chung and Subbiah, 1996; Do *et al.*, 2006; Jaroszewski *et al.*, 2000; Rost, 1999; Sumanaweera *et al.*, 2019). Subsequent studies have extended this metaphor to suggest the term *midnight zone* of sequence relationships, where the pairs of sequences being aligned have mutated beyond any feasible recognition using sequence information alone (Bujnicki, 2003; Meier and Söding, 2015; Reid *et al.*, 2007).

Therefore, to reliable use pairwise sequence alignments, it is necessary to understand their ‘safe operating ranges’ and the natural limits of their inference. That is, understand when the sequence and structure alignments of pairs produce consistent answers (the ‘daylight’ zone of protein sequence relationships), when the reliability of sequence alignments gives diminishing returns compared to structures (the twilight zone) and hence handled with caution for subsequent downstream studies, and finally when the evidence of residue–residue relationships become untenable and sequence-based alignments are best ignored (the midnight zone).

Chothia and Lesk (1986) and Rost (1999) discuss the relationship between divergence of sequence and structure. Chothia and Lesk (1986) were the first to quantify this relation. Based on their concept of the ‘common core’ between pairs of protein structures, they fit a curve with an ability to predict the root-mean-square deviation (rmsd) of least-squares superposition of common cores from the fraction *f* of amino acids strictly conserved within the core, that generalizes to domains of different structural types: rmsd=0.4×exp(1.87(1−f)).

Rost (1999), in his subsequent analysis of a larger data set, relied on the use of the Smith-Waterman method (and BLAST) with ‘fixed’ sequence alignment parameters and a binary {true, false} notion of structure relationship. He did *not* consider the relationship between divergence of sequence and structure at a residue level. Nevertheless, Rost (1999) showed that the ability to distinguish, coarsely, between similar and non-similar pairs of protein structures diminished (unsurprisingly) as sequence identity decreased, with an explosion of false positives when the sequence identity between proteins fell between [20%, 35%].

A more thorough assessment of the consistency of residue–residue relationships derived from sequences and structures requires modeling the deteriorating quality of sequence alignments as proteins diverge. Further, the utility of any systematic assessment is enhanced if it stands on a sound mathematical foundation and is based on effective and consistent statistical models, whose parameters are not prespecified or frozen, but rather automatically inferred as a part of the statistical framework of comparison.

This issue motivates our current work, which attempts to study more systematically how sequences and structures diverge in evolution using, to the best of our knowledge, techniques not previously explored. We build on top of our recently developed Bayesian framework for protein sequence comparison (Sumanaweera *et al.*, 2019), which uses the powerful statistical inductive inference criterion of Minimum Message Length (MML) (Allison, 2018; Wallace, 2005; Wallace and Boulton, 1968). This framework infers automatically (i.e. unsupervised) how far the sequences have diverged, by statistically estimating the ‘time’ parameter associated with the underlying stochastic Markov matrix of amino acid substitutions. This (Markov) time parameter can be converted to a theoretical *expectation* of the fraction of amino acids that can change under that model of relationship. Further, it provides a powerful method to decipher distant sequence relationships between proteins when no *singular* alignment relationship is significant, by computing the *marginal probability* over all possible alignments between the sequence pair, to adjudicate their relationship (see Section 2).

Building on this framework, the current work introduces a natural and intuitive distance measure between any two given alignments for any protein pair. Here, we avoid using this measure of distance to compare a single *biased* sequence alignment against its structural counterpart. Instead, we compute the exact *mathematical expectation* of the distance between sequence and structure alignments, by *integrating* over all possible sequence alignments for any given protein pair, weighted by their individual *marginal* probabilities.

Note that despite the number of total possible alignments between any pair of sequences growing factorially large as a function of their sequence lengths, we provide an exact quadratic-time algorithm (in sequence length) to calculate this mathematical expectation of the inter-alignment distance between sequence and structure alignments, using probabilities estimated by our Bayesian and MML framework.

Further, to validate the above exact computation, we also compute an empirical expectation of the distance to compare against. This empirical expectation is achieved by randomly sampling not a single, but thousands of alignments over the marginal probability matrices that are generated as a part of the MML framework.

Finally, we evaluate the sequence and structure alignments of *1 million* pairs of protein domains and analyze their lossless compression statistics, the inferred (Markov) time parameter of sequence divergence, the expected fraction of amino acid changes and the expected and empirical inter-alignment distance with respect to their reference structure alignments. Our analysis reveals the distinct ranges of amino acid divergences where pairwise sequence alignments could be trusted, the ranges where they should be handled with caution, and those where they should be ignored.

## 2 Background

We briefly summarize the main features of the protein sequence comparison framework (Sumanaweera *et al.*, 2019) central to this work.

### 2.1 MML framework

The MML criterion (Allison, 2018; Wallace, 2005; Wallace and Boulton, 1968) is a Bayesian and information-theoretic method of unsupervised parameter estimation and hypothesis selection. This method can be understood as a lossless communication process between an imaginary transmitter and receiver. The goal of the communication is to transmit the observed data *D* losslessly and economically to the receiver. The transmission comes in two parts: the first explains the hypothesis *H* on the data, the lossless encoding length of which is denoted as I(H); the second explains the details of the data *D* not already covered by the hypothesis (which was already been communicated in the first part), the lossless encoding length of which is denoted by I(D|H). This two-part message length can thus be formalized as

I(H,D)=I(H)+I(D|H)=I(D)+I(H|D).

From the Bayesian point of view, the two-part message length *I*(*H*, *D*) is an information-theoretic restatement of the product axiom of probability, Pr(H,D)=Pr(H)Pr(D|H)=Pr(D)Pr(H|D), after applying Shannon’s measure of information content (Shannon, 1948), where the optimal bound on the length of the lossless encoding of any event *E* with a probability Pr(E) is given by I(E)=−log(Pr(E)). Note that when the logarithm is in base-2, the length is measured in *bits*.

The MML criterion can therefore be used to compare any two competing hypotheses, *H* and H′, for explaining the same data *D*, since the difference in their message lengths of lossless encoding, I(H,D)−I(H′,D), gives a log-odds posterior ratio test of their relative significance:

$$I(H,D)-I(H\prime ,D)=-\text{log}\;\left(\frac{\mathrm{Pr}(D)\mathrm{Pr}(H|D)}{\mathrm{Pr}(D)\mathrm{Pr}(H\prime |D)}\right)=\text{log}\;\left(\frac{\mathrm{Pr}(H\prime |D)}{\mathrm{Pr}(H|D)}\right).$$

Therefore, over the space of all possible hypotheses **H**, a best hypothesis $H^{*}\in H$ is one that gives the *minimum* value for $I(H^{*},D)$:

$$H^{*}=\arg{\underset{\forall H\in H}{\min}}_{}I(H)+I(D|H).$$

This defines the MML criterion for hypothesis selection.

Further, implicit within the MML framework is a natural *null hypothesis test*. This test relies on the null model message, i.e. the efficient lossless encoding of the data *D* without the aid of any hypothesis, the length of which is denoted by NULL(D). If any hypothesis *H* does not beat the null model message length, i.e. if NULL(D)−I(H,D)≤0 then *H* is *rejected*, else it is *accepted*.

### 2.2 Protein sequence comparison using MML

Sumanaweera *et al.* (2019) describe an MML framework for protein sequence comparison. Any alignment A between a pair of amino acid sequences 〈S,T〉 specifies a hypothesis of their residue–residue relationship. The complexity of this alignment relationship [denoted by I(A)] together with its fidelity to explain losslessly all the observed amino acids within the sequence pair (I(〈S,T〉|A)) can be used to differentiate between hypotheses and select an optimal one. Specifically, the first part of the message losslessly encodes the alignment as a three-state string generated from a (Markov) time-parameterized finite-state machine over three states: match, insert and delete. The second part of the message then encodes losslessly the details of the amino acid symbols of the sequences *S* and *T* with the aid of the alignment relationship A specified in the first part, while using a (Markov) time-parameterized stochastic matrix to encode amino acids corresponding to the matched columns in A and a 20-nomial model to encode amino acids that are unmatched. An optimal alignment (and its internal time parameter) is automatically inferred by minimizing the two-part message length

(1) $$I(A,\langle S,T\rangle )=\underset{\text{First part}}{\underset{︸}{I(A)}}+\underset{\text{Second part}}{\underset{︸}{I(\langle S,T\rangle |A)}}\;\;\text{bits}.$$

Recall, from the general introduction to MML above, that the two-part message length of any alignment is the negative logarithm of the joint probability of the alignment and the sequences

$$I(A,\langle S,T\rangle )=-\text{log}(\underset{\text{Joint probability}}{\underset{︸}{\mathrm{Pr}(A,\langle S,T\rangle )}}).$$

Further, the framework described in Sumanaweera *et al.* (2019) also allows the estimation of the *marginal probability* $\underset{\text{marginal}}{\mathrm{Pr}}(\langle S,T\rangle )$  *of the two sequences being related*. Importantly, this estimation of marginal probability provides a reliable way to identify a distant relationship between sequences even when any single alignment A (even an optimal alignment, $A^{*}$) is *not* statistically significant. That is, in the MML framework, this implies I(A,〈S,T〉)≥NULL(〈S,T〉)=NULL(S)+NULL(T).

The marginal probability is estimated by summing the joint probabilities Pr(A,〈S,T〉) over all possible alignments (∀A∈A) between the sequence pair 〈S,T〉:

(2) $$\underset{\text{marginal}}{\mathrm{Pr}}(〈S,T〉)=\sum_{\forall A\in A}\mathrm{Pr}(A)\mathrm{Pr}(〈S,T〉|A)$$

and its negative logarithm is denoted as

(3) $$I_{\text{marginal}}(\langle S,T\rangle )=-{\;\text{log}\;}_{2}\left(\underset{\text{marginal}}{\mathrm{Pr}}(\langle S,T\rangle )\right)\;\text{bits}.$$

Note that the number of all possible alignments A∈A between any sequence pair 〈S,T〉 grows factorially as a function of the sequence lengths (denoted by |S| and |T|) as follows:

$$|A|=\sum_{\left|A|=\max(|S|,|T|\right)}^{\left|S|+|T\right|}\frac{|A|!}{(|A|-|S|)!(|A|-|T|)!(|S|+|T|-|A|)!}.$$

Despite this growth, the estimation of marginal probability can be implemented over a two-dimensional dynamic programming algorithm that runs in time proportional to O(|S||T|), as described in Sumanaweera *et al.* (2019).

The program implementing the above MML framework for pairwise sequence comparison, seqMMLigner, allows the efficient computation of an optimal alignment $A^{*}$ of any given sequence pair [by minimizing Equation (1)] and also estimates the marginal probability that the pair is related [Equation (3)]. In doing this, seqMMLigner yields key statistics on the relationship between the two sequences. Specifically, when computing an optimal alignment $A^{*}$, the program also reports its lossless encoding length $I(A^{*},\langle S,T\rangle )$, the amount of compression gained (>0) or lost (≤0) with respect to the null encoding via $\Delta_{\text{optimal}}=\text{NULL}(\langle S,T\rangle )-I(A^{*},\langle S,T\rangle )$, and a *biased* Markov time parameter $\mathrm{tim}e_{\text{optimal}}$ which gives an estimate of the inferred divergence of any sequence pair based on the *single* optimal alignment. When estimating the marginal probability of the two sequences being related, the program also reports the amount of compression gained (>0) or lost (≤0) with respect to the null encoding via $\Delta_{\text{marginal}}=\text{NULL}(\langle S,T\rangle )-I_{\text{marginal}}(\langle S,T\rangle )$, an *unbiased* Markov time parameter $\mathrm{tim}e_{\text{marginal}}$ which considers all possible alignment relationships between the two sequences, and a marginal probability landscape (e.g. refer to the landscape plots in Section 4, Figure 4). Each landscape is denoted by an ((|S|+1)×(|T|+1))-order matrix, where each cell (i,j),∀0≤i≤|S|,0≤j≤|T|, stores the negative logarithm of the *product of marginal probabilities* of their prefixes $\langle S_{1\ldots i},T_{1\ldots j}\rangle$ being related (i.e. $\underset{\text{marginal}}{\mathrm{Pr}}(\langle S_{1\ldots i},T_{1\ldots j}\rangle )$), and their suffixes $\langle S_{i\ldots |S|},T_{j\ldots |T|}\rangle$ being related (i.e. $\underset{\text{marginal}}{\mathrm{Pr}}(\langle S_{i\ldots |S|},T_{j\ldots |T|}\rangle )$).

These key statistics form the basis of the methods presented in our current work.

## 3 Materials and methods

### 3.1 Distance between alignments

Considering a very large data set of homologous protein domain pairs with known sequences and structures, our aim is to quantify the qualitative differences between sequence alignments and their structural counterparts. At a fundamental level, this requires some reliable measure of *distance* between two alignments (in our case, a sequence alignment and the reference structure alignment). The most basic measure of distance between two alignments can be obtained by counting the fraction of columns in the two alignments that are *concordant* with each other, i.e. the fraction of identically aligned columns (including insertions and deletions) between them. However, this distance measure is not very informative as it only provides a binary yes (‘columns are identical’) or no (‘columns are not identical’) measure of distance, which can render very closely related alignments as fully distant from each other.

To overcome this issue, we define below a better and more intuitive way to measure the distance between any two alignments for any protein pair. We then use this measure to compute the *mathematical expectation* of the distance between the sequence and structure alignments over *all* possible alignment relationships between the two amino acid sequences, weighted by their *posterior probabilities*.

#### 3.1.1 Inter-alignment distance

**Fig. 1.**
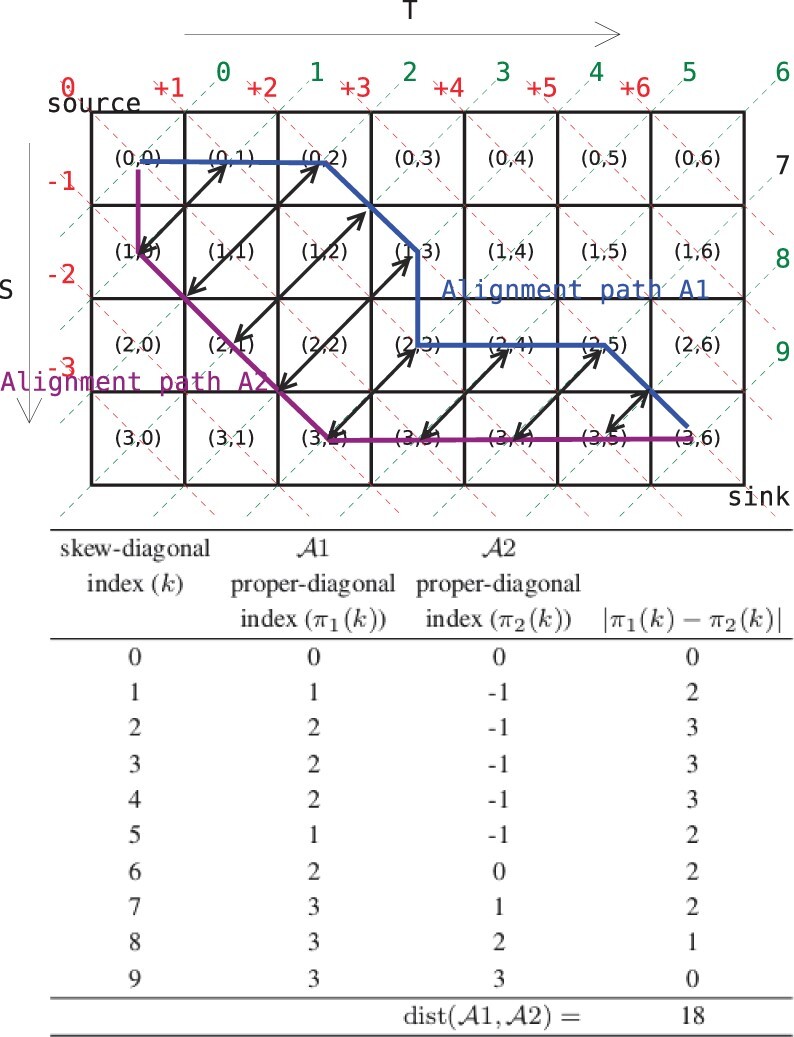
An illustration of the distance between two alignments measured as the area between two source-to-sink paths

Any alignment between two proteins can be expressed as a source-to-sink path in a matrix indexed by their sequences. Figure 1 illustrates two alignment source-to-sink paths. Notice that at each cell (*i*, *j*), any alignment path traverses either horizontally to the right [i.e. (i,j)→(i,j+1)], vertically below [i.e. (i,j)→(i+1,j)] or diagonally across [i.e. (i,j)→(i+1,j+1)]. Therefore, a natural measure of distance between alignments can simply be expressed as a function of the space (or area) between the two paths. This not only captures all the identities in the alignments, but also their ‘closeness’ when columns between the two alignments are not identical.

For ease of computation, we quantify this space as follows. As shown on the upper section of Figure 1, each alignment path crosses each skew diagonal of the matrix (indexed in green) exactly once. This is exploited to define the distance between two alignment paths as a summation of the width between the two alignments at each skew diagonal, where the width is simply the absolute difference in the corresponding proper-diagonal indices (in red) of the matrix through which the alignment paths pass.

The lower section of Figure 1 shows a worked out example for this computation. For each of the two alignments A1 and A2, the skew-diagonal indices run in the range [0,|S|+|T|]—for any cell (*i*, *j*), its skew-diagonal index is given by *i *+* j*. The signed proper-diagonal index fall in the range [−|S|,+|T|]—for any cell (*i*, *j*), its corresponding proper-diagonal is given by *j* − *i*. If $\pi_{k}(\cdot )$ defines a mapping from a skew-diagonal index (that the alignment path cuts through) to its corresponding proper-diagonal index for an alignment Ak, then the measure of distance between the two paths of alignments A1 and A2 can be formulated as

(4) $$\text{distance}(A1,A2)=\sum_{k=0}^{\left|S|+|T\right|}\delta (k)=\sum_{k=0}^{\left|S|+|T\right|}|\pi_{1}(k)-\pi_{2}(k)|.$$

### 3.2 Expected inter-alignment distance with respect to a reference alignment

Since the reliability of any single sequence alignment between two protein sequences deteriorates as the proteins diverge, we propose here a novel extension to the way the alignment distance can be estimated using the MML framework.

Instead of measuring the distance of any *single* (optimal) sequence alignment with respect to its structure alignment, we propose the computation of the *mathematical expectation* of the inter-alignment distance (relative to a reference structure alignment) weighted by the posterior probabilities of all sequence alignments between the two proteins.

Let **A** denote the set of all possible alignments between the pair of sequences 〈S,T〉. The expected inter-alignment distance with respect to any reference alignment $A_{\text{ref}}$ can be formulated as

(5) $$E[\text{distance}(A,A_{\text{ref}})]=\sum_{\forall A\in A}\underset{\text{posterior probability}}{\underset{︸}{\mathrm{Pr}(A|\langle S,T\rangle )}}\times \text{distance}(A,A_{\text{ref}}),$$

where the posterior probability is the joint probability normalized by the marginal: $\mathrm{Pr}(A|\langle S,T\rangle )=\mathrm{Pr}(A,\langle S,T\rangle )/\underset{\text{marginal}}{\mathrm{Pr}}(\langle S_{1\ldots i},T_{1\ldots j}\rangle )$.

Note that we need to calibrate the resulting expected distances to permit comparison across sequence pairs with different lengths. An approach is to divide $E[\text{distance}(A,A_{\text{ref}})]$ by the product of their lengths, |S||T| (i.e. worst-case distance). Although this bounds the distance between [0,1], in practice this divisor dominates the observed values, making it difficult to discriminate distances across pairs. To make the comparison more intuitive our analysis uses as divisor the sum of the sequence lengths, |S|+|T|, which is the maximum possible length of any alignment.

#### 3.2.1 Computation of the expected inter-alignment distance using a two-dimensional dynamic programming algorithm

Although the cardinality of the set of all possible alignments grows factorially in the lengths of the sequences, the computation of the mathematical expectation can be achieved in both time and space that is proportional to O(|S||T|).

Three matrices denoted by $EAD^{m},\;EAD^{i}$ and $EAD^{d}$ of order ((|S|+1)×(|T|+1))  *memoize* the expected distance statistic of all possible *subproblems*. Specifically, any cell (i,j),∀0≤i≤|S|,0≤j≤|T|, across each of the three matrices stores the expected distance over all possible sequence alignments of the prefixes of the two sequences $\langle S_{1\ldots i},T_{1\ldots j}\rangle$ ending at the cell (*i*, *j*) in a match (m), insert (i) or delete (d) states, respectively, weighted by their unnormalized joint probabilities.

By exploiting the Bellman condition for this problem, the following dynamic programming recurrence can be formulated over the three memoized subproblem matrices (see Supplemenary Section S1 for a derivation of these recurrences)

(6) $$\begin{array}{c} EAD^{m}(i,j)=EAD^{m}(i-1,j-1)\mathrm{Pr}(m|m)\mathrm{Pr}(s_{i},t_{j})\\ +EAD^{i}(i-1,j-1)\mathrm{Pr}(m|i)\mathrm{Pr}(s_{i},t_{j})\\ +EAD^{d}(i-1,j-1)\mathrm{Pr}(m|d)\mathrm{Pr}(s_{i},t_{j})\\ +\underset{\text{marginal}}{\mathrm{Pr}}(\langle S_{1\ldots i},T_{1\ldots j}\rangle |\;\mathrm{match}@(i,j))\times \left[\begin{array}{c} \delta (i+j-1)\\ +\delta (i+j) \end{array}\right] \end{array},$$

 

(7) $$\begin{array}{c} EAD^{i}(i,j)=EAD^{m}(i-1,j)\mathrm{Pr}(i|m)\mathrm{Pr}(s_{i})\\ +EAD^{i}(i-1,j)\mathrm{Pr}(i|i)\mathrm{Pr}(s_{i})\\ +EAD^{d}(i-1,j)\mathrm{Pr}(i|d)\mathrm{Pr}(s_{i})\\ +\underset{\text{marginal}}{\mathrm{Pr}}(\langle S_{1\ldots i},T_{1\ldots j}\rangle |\;\mathrm{insert}@(i,j))\times \delta (i+j) \end{array},$$

 

(8) $$\begin{array}{c} EAD^{d}(i,j)=EAD^{m}(i,j-1)\mathrm{Pr}(d|m)\mathrm{Pr}(t_{j})\\ +EAD^{i}(i,j-1)\mathrm{Pr}(d|i)\mathrm{Pr}(t_{j})\\ +EAD^{d}(i,j-1)\mathrm{Pr}(d|d)\mathrm{Pr}(t_{j})\\ +\underset{\text{marginal}}{\mathrm{Pr}}(\langle S_{1\ldots i},T_{1\ldots j}\rangle |\;\mathrm{delete}@(i,j))\times \delta (i+j) \end{array}.$$

In the recurrence 6, the term $\underset{\text{marginal}}{\mathrm{Pr}}(\langle S_{1\ldots i},T_{1\ldots j}\rangle |\;\mathrm{match}@(i,j))$ denotes the component marginal probability of all alignments of the prefixes of the two sequences ending in a match at the cell (*i*, *j*). (Similar definitions hold the other two component marginal probability terms in 7–8.) Running seqMMLigner in its marginal mode generates three matrices (one for each state) containing these component marginal probability terms. In other words, the marginal probability matrices of the two sequences as described in Sumanaweera *et al.* (2019) are first computed, before the dynamic program shown above is run. The terms Pr(m|m),Pr(d|m),Pr(i|m) in the recurrence 6 are the respective probabilities of a (Markov) time-parameterized three-state alignment machine transitioning from {match, insert, delete} states in the cell (i−1,j−1) to a match state in the cell (*i*, *j*), using the inferred $\mathrm{tim}e_{\text{marginal}}$. (Similar terms in recurrences 7–8 deal with respective transitions to insert and delete states.)

The $\mathrm{Pr}(s_{i},t_{j})$ term in recurrence 6 gives the joint probability of the pair of amino acids being in the match state in the alignment. This probability comes from the underlying time-parameterized stochastic Markov matrix of amino acid interchanges, at the inferred $\mathrm{tim}e_{\text{marginal}}$. On the other hand, the terms $\mathrm{Pr}(s_{i})$ and $\mathrm{Pr}(t_{j})$ in recurrence 7–8 are the time-*independent* 20-nomial probabilities of amino acids, accounting for the inserted and deleted amino acids, respectively.

Finally, the δ(·) terms across the three recurrences denote the width element along any specified skew-diagonal index (see Figure 1) in the computation of distance, similar to the one used in Equation (4). In the recurrences above, at each cell (*i*, *j*) one is accounting for the additional new widths so that the subproblems at (i−1,j−1), (i−1,j) and (i,j−1) can grow to any (*i*, *j*). Notice that in recurrence 6, two δ(·) terms are involved, as all alignments going between the cells (i−1,j−1)→(i,j) [i.e. landing in (*i*, *j*) in a match state] cuts across two (not one) skew diagonals. Hence, both widths, one for the skew-diagonal index i+j−1 and other for the index *i *+* j*, need accounting for in the dynamic program.

The above dynamic program can be computed bottom-up starting from the trivial subproblem where the expected inter-alignment distance at the source is 0: $EAD^{m}(0,0)=EAD^{i}(0,0)=EAD^{d}(0,0)=0$. Once the matrices get filled, the expected inter-alignment distance defined in Equation (5) is computed by summing $EAD^{m}(|S|,|T|)+EAD^{i}(|S|,|T|)+EAD^{d}(|S|,|T|)$ and dividing it by the marginal probability. Since the marginal probability matrices are precomputed (in O(|S||T|) space/time) before running the above dynamic program, and all other terms at each cell (*i*, *j*) can be evaluated in *O*(1) time, it is straightforward to see that the time and space complexity of the computation of this mathematical expectation is O(|S||T|).

Based on the method of computation presented here, seqMMLigner (v2.5) has been extended for users to be able to compute the expected distance between sequence and any given reference alignment.

## 4 Results and discussion

We randomly sampled *1 million* distinct pairs of protein domains across four major structural classes of SCOPe (v2.07): all-*α*, all-*β*, *α*/*β* and *α *+ *β*. To restrict the analysis to pairs of homologous proteins, the domains in each sampled pair were drawn from either the same ‘superfamily’ (yielding 541 162 domain pairs) or same ‘family’ (yielding 458 838 domain pairs) as defined by their SCOP classification (Murzin *et al.*, 1995). (The list of domain pairs is available from the Supplementary Section S2.)

Table 1 gives the distribution of the sampled domain pairs across four SCOP classes. It further shows the number of those pairs for which: (i) their optimal alignments are statistically significant ($\Delta_{\text{optimal}}>0$), (ii) their marginal probability detects the sequence relationship, but their optimal alignments do not ($\Delta_{\text{optimal}}\leq 0<\Delta_{\text{marginal}}$) and (iii) when neither the optimal alignments nor the marginal probabilities are able to provide evidence of a relationship solely from the sequence information ($\Delta_{\text{marginal}}\leq 0$), but their relationship is ascertained from their SCOP structure classification and structural alignment. Together, the considered domain pairs set covers the full spectrum of possible sequence-divergence between protein pairs, and is thus useful to analyze systematically the limits of inferring sequence alignment relationships, and to identify the ‘daylight’, ‘twilight’ and ‘midnight’ zones of sequence relationships among homologous proteins.

**Table 1.** Distribution of the sampled 1 million domain pairs, subdivided into three distinct groups/subsets based on their optimal and marginal compression statistics measuring the significance of sequence relationships (see Section 2.2)

SCOP class	SCOP level	Compression-based distinct group sizes		
		$\Delta_{\text{optimal}}>0$	Δ_optimal_ ≤ 0 and Δ_marginal_ > 0	$\Delta_{\text{marginal}}\leq 0$
All-*α*	Family	90 710	25 093	18 043
	Superfamily	23 607	35 187	44 346
All-*β*	Family	59 962	12 250	14 020
	Superfamily	30 810	23 869	117 717
*α*/*β*	Family	35 437	22 456	12 803
	Superfamily	25 087	82 169	76 743
*α *+ *β*	Family	112 840	48 687	6537
	Superfamily	38 631	24 233	18 763

The structures of all 1 million domain pairs were separately aligned using three structure alignment programs: (i) (structure) MMLigner (Collier *et al.*, 2017), (ii) TM-align (Zhang and Skolnick, 2005) and (iii) DALI (Holm and Sander, 1995). These provide three distinct sets of reference structure alignments ($A_{\text{ref}}$) against which the expected distance of the sequence alignment will be computed. In addition, the sequences of all domain pairs were compared using seqMMLigner (v2.5) (Sumanaweera *et al.*, 2019) under both its marginal and optimal modes. To evaluate the effect of changing the time-parameterized models used in this MML framework, each domain pair was compared using four different stochastic Markov matrices and their corresponding three-state machine models derived by Sumanaweera *et al.* (2020). The time-parameterized models in this analysis cover BLOSUM (Henikoff and Henikoff, 1992), VTML (Müller *et al.*, 2002), MMLSUM (Sumanaweera *et al.*, 2020) and PAM (Dayhoff *et al.*, 1978).

Using the methodology presented in Section 3, we computed the expected distance of sequence alignments and the inferred Markov time parameter ($\mathrm{tim}e_{\text{marginal}}$) for each of the million domain pairs, over all possible combinations of the four time-parameterized models and the three reference structure alignments considered. (See Supplementary Website for the raw data from these runs.)

Each of these 12 possible combinations generated a million data points, respectively. These data points can be grouped into bins based on the inferred Markov time ($\mathrm{tim}e_{\text{marginal}}$) of each comparison, in the range [1, 500]. This time-range corresponds to the expected %-change of amino acids in the range of [1%,∼92%].

**Fig. 2.**
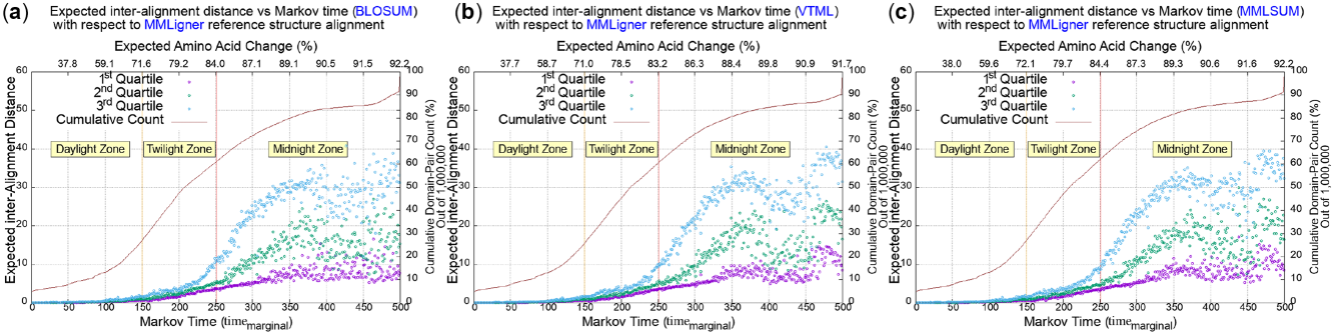
First, second and third quartile statistics of the expected distance of sequence alignments with respect to a reference alignment obtained using MMLigner (Collier *et al.*, 2017) program as a function of the inferred Markov time parameter, $\mathrm{tim}e_{\text{marginal}}$. The three columns correspond to the three time-parameterized models employed during the MML sequence comparison: (a) BLOSUM, (b) VTML and (c) MMLSUM (Sumanaweera *et al.*, 2020). In each plot, the *x*-axis (shown below the box) is the Markov time step in the range [1, 500]; the range on the top of the plot is the corresponding expected %-change of amino acids for the chosen time-parameterized model; the *y*-axis (left of the box) is the expected distance; the scatter plots (magenta, green and blue) track the changes in the first, second and third quartile statistics of the expected distance statistic over 1 million domain pairs, grouped according to their inferred integer Markov time step ($\mathrm{tim}e_{\text{marginal}}$); the vertical range on the right of the box tracks the cumulative %-growth of the number of domain pairs as a function of time on the *x*-axis

For all the domain pairs that group at each inferred integer $\mathrm{tim}e_{\text{marginal}}$ bin in the range [1, 500], their first (Q1), second (Q2) and third (Q3) quartile statistics of the expected distances are tracked. Figure 2 presents the variation of the expected distance of alignments versus $\mathrm{tim}e_{\text{marginal}}$. (The plots corresponding to the PAM model for sequence, DALI and TM-Align for structure comparisons are available in the Supplementary Section S3.1.)

Analyzing the plots shown in Figure 2, a consistent pattern emerges, *independent* of the time-parametrized models and reference alignments employed. For all domain pairs with inferred $\mathrm{tim}e_{\text{marginal}}\leq 150$, their sequence and structure alignments agree very closely, as can be seen from the low value of the computed expected distance as well as the low variance between the quartile trends for each time step in the range. This time-range corresponds to pairs of sequences that have undergone ≤71% (expected) change in amino acids, as shown by the scale on the top of each plot. Further, the $\mathrm{tim}e_{\text{marginal}}$ range of [1, 150] accounts for only ∼25% of the domain pairs sampled, as seen from the cumulative growth curves (the continuous curve shown in brown in each plot, whose vertical scale appears on the right side of each plot). This time-range of [1, 150] thus denotes the *daylight* zone of inference of sequence alignments, which are highly consistent with those inferred from structures.

In the time-range of [150, 250], the expected distance as well as the variance between the quartiles ramps up steeply. This region corresponds to an expected amino acid %-change of [∼71%−∼84%], and accounts for a further ∼35% of the domain pairs sampled. This specifies the *twilight* zone of inference of sequence alignments. The degree of belief in any specific residue–residue correspondence in this range becomes unreliable. By selecting pairs of sequences and examining their optimal sequence alignments and corresponding structural alignments, we observe that the differences in residue–residue correspondences correlate more with the peripheral regions of the domains, than with the relationships defined between residues in the buried core of the structures, especially in the time-range of [150, 200] (or equivalently [∼71%−∼79%] expected %-change in amino acids). However, in the second half of the twilight zone (inferred time between [200–250]), the differences spread out even to the buried core and hence the sequence alignments become more and more unreliable in the twilight zone.

Finally, we observed that the *midnight* zone of protein sequence relationships, where the inferred $\mathrm{tim}e_{\text{marginal}}>250$, is marked by a drastic growth in the variance between the quartiles, as seen across plots in the time-range [250–350]. This corresponds to a region where sequences undergo an expected amino acid change of [∼84%−∼89%] and account for about 20% of the total domain pairs considered. Beyond this (time > 350), the quartile scatter plots become extremely noisy. Thus, it is prudent to conclude that for the inferred time > 350 (or equivalently  89% expected %-change of amino acids) the sequences have diverged beyond the very limit of inference of pairwise sequence alignments.

**Fig. 3.**
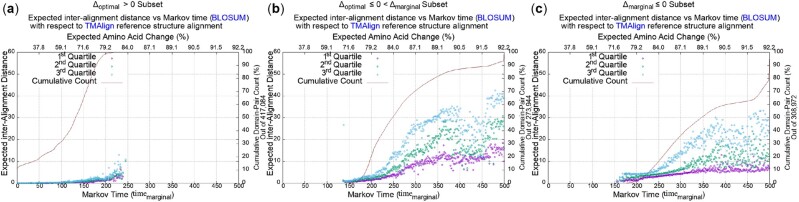
Expected distance versus Markov time plots, after separating the million domain pairs into distinct groups based on their compression statistics—see Table 1. (a) The variation of expected distance of sequence alignment on the subset of the domain pairs where the optimal alignment model beats the null ($\Delta_{\text{optimal}}>0$). (b) Same as above, but corresponding to the subset of the domain pairs for which the marginal beats the null but not the optimal ($\Delta_{\text{optimal}}\leq 0<\Delta_{\text{marginal}}$). (c) As above, but for the remaining domain pairs where neither the optimal nor the marginal beats the null

Complementing the above observations of the daylight, twilight and midnight zones, is the analysis we performed based on the subsets/groups defined by the respective statistical significance test statistics ($\Delta_{\text{optimal}}$ and $\Delta_{\text{marginal}}$). Figure 3 shows three plots that divide the million domain pairs into three groups. (See last three columns of Table 1.) The first group accounts for 417 084 domain pairs whose optimal sequence alignment under the MML framework yielded positive compression (i.e. $\Delta_{\text{optimal}}>0$). The second group accounts for 273 944 domain pairs whose optimal sequence alignment lost to the null model but gave positive compression using the marginal model (i.e. $\Delta_{\text{optimal}}\leq 0<\Delta_{\text{marginal}}$). The final group contains the remaining 308 972 domain pairs where $\Delta_{\text{marginal}}\leq 0$.

Figure 3a shows that ∼70% of the 417 084 domain pairs are in the daylight zone (i.e. $\mathrm{tim}e_{\text{marginal}}\in [1,150]$), and another 28% fall within the first half of the twilight zone [150, 200], a region where the common cores are reliably aligned from sequences alone, as observed above. This is consistent with the observation that all pairs in this group yielded $\Delta_{\text{optimal}}>0$, indicating their single alignments are reliable. Figure 3b shows that ∼50% of the 273 944 domain pairs fall within the twilight zone (time between [150, 250]), and the remaining 50% in the midnight zone region ≥250. This is consistent with the fact that no single alignment (including optimal) is statistically significant in this group (i.e. $\Delta_{\text{optimal}}\leq 0$), but the marginal probability over all possible alignments is able to detect the relationship in some unspecified way (i.e. $\Delta_{\text{marginal}}>0$). Finally, Figure 3c shows that >80% of the domain pairs for which even the marginal probability cannot assert a relationship, fall in the midnight zone, with inferred Markov time > 250. Our analysis shows that this pattern of relationship is not affected by changes to the time-parameterized models or to the structural alignment programs (see Supplementary Section S3.2 for the plots corresponding to the other models and programs).

Next, we verify the computation of the mathematical expectation of distance, using an empirical (sampling) approach that can be used to generate highly probable alignments using the marginal probability matrices generated by seqMMligner. For each of the million domain pairs, 1000 alignments were sampled from the marginal probability matrix of each pair. The distance between each sampled alignment for a pair was compared to its corresponding reference structure alignment using Equation (4). The resulting estimates of distance were then averaged to generate an *empirical* estimate of the distance that can be compared (as a consistency-check) against the exact mathematical expectation computed by Equation (5). This provides a way to validate the expected distances we compute and report. (See the Supplementary Section S3.3 for details and plots.)

Further, to better understand the correlations between the SCOP classification of domains and the identified ranges for *daylight*, *twilight* and *midnight* zones, we sampled >200 000 distinct domain pairs related at varying levels of SCOPe (v2.07). This yields five distinct groups of domains pairs with their pairwise SCOP relationship similar up to the level of (i) same ‘family’ (yielding 55,108 domain pairs), (ii) same-‘superfamily’ (yielding 31,585 pairs), (iii) same ‘fold’ (yielding 40,530 pairs), (iv) same ‘class’ (yielding 40,498 pairs) and (v) ‘decoy’ (i.e. different class, yielding 40,416 pairs)—see Supplementary Table S1. As before, we compute the expected inter-alignment distance and infer Markov time parameter ($\mathrm{tim}e_{\text{marginal}}$) for each dataset of domain pairs using MMLSUM time-parameterized models and reference structure alignments generated from MMLigner.

Figure S6 (in Supplementary Section S4) shows that the daylight zone of sequence relationship accounts for ∼63% of domain pairs sampled at the ‘same-family’ level and ∼27% of domain pairs sampled at the ‘same-superfamily’ level. As expected, 0% of the domain pairs sampled at the levels of ‘same-fold’, ‘same-class’ and ‘decoy’ fall in the daylight zone.

Next, the twilight zone accounts for ∼25% of pairs sampled at the ‘same-family’ level, ∼28% of those sampled at ‘same-superfamily’ level and only ∼1% at the level of ‘same-fold’ and ‘same-class’, while it remains 0% for the ‘decoy’ set.

Furthermore, the midnight zone accounts for ∼12% of pairs at the ‘same-family’ level, ∼45% at the ‘same-superfamily’ level and ∼99% at the levels of ‘same-fold’ and ‘same-class’. Nearly 100% of the domain pairs in the ‘decoy’ set fall in the midnight zone.

These numbers reveal a broad agreement with the classification criterion used in SCOP, where related proteins with similar sequences fall under the same family and proteins with weak sequence similarity signal, but detectable functional and structural conservation is classified under same superfamily. Similar superfamilies without compelling evidence of common evolutionary origin are grouped into folds, which are further arranged into classes mainly encompassing the recurrent secondary structural features and their architecture. As a future exercise, it would be useful to examine the domain pairs that fall in the midnight zone of sequence relationship, despite being related at the same-family or same-superfamily levels.

Finally, we present a qualitative case study comparing the sequence of human hemoglobin (1HHO chain A) with the sequences of six other related proteins, at varying levels of sequence divergence, but all classified within the same SCOP Globin-like SCOP fold: (i) chicken hemoglobin (1HBR chain A), (ii) sperm whale myoglobin (1MBD), (iii) *Chironomus* erythrocruorin (1ECA chain A), (iv) bacterial hemoglobin (4VHB chain A), (v) paramecium truncated hemoglobin (1DLW chain A) and (vi) red-alga phycocyanin (2BV8 chain A).

Table 2 presents the following key statistics inferred for pairwise sequence comparison of each of the above proteins with human hemoglobin: the message length terms over the marginal, optimal and null models ($I_{\text{marginal}}(\langle S,T\rangle )$, $I(A^{*},\langle S,T\rangle )$ and NULL(〈S,T〉)), the inferred unbiased estimate of the time parameter ($\mathrm{tim}e_{\text{marginal}}$) with respect to the marginal model, its corresponding expected amino acid change, and the expected inter-alignment distance.

**Table 2.** Key statistics inferred from the MML framework [using seqMMLigner (v2.5) (Sumanaweera *et al.*, 2019)], comparing the sequences of a set of six homologous domains from the Globin-like folds with the *α* chain sequence for human hemoglobin (1HHO chain A)

*S* versus *T*	$I_{\text{marginal}}(\langle S,T\rangle )$ (bits)	$I(A^{*},\langle S,T\rangle )$ (bits)	NULL(〈S,T〉) (bits)	Inferred time	Exp. a.a. change (%)	E.A.D.
Human [1HHO(A)] versus chicken [1HBRA(A)]	940.4	940.7	1186.9	49	37.5	0.006
Human [1HHO(A)] versus sperm whale (1MBD)	1198.8	1203.0	1241.6	157	73.4	0.374
Human [1HHO(A)] versus *Chironomus* [1ECA(A)]	1166.7	1179.1	1174.4	198	79.4	1.202
Human [1HHO(A)] versus bacterium [4VHB(A)]	1165.6	1177.1	1173.8	226	82.4	1.456
Human [1HHO(A)] versus paramecium [1DLW(A)]	1082.3	1097.0	1087.9	235	83.2	2.776
Human [1HHO(A)] versus red-alga [2BV8(A)]	1277.9	1299.8	1277.3	307	87.7	15.415

The rows of this table appear in the increasing order of the resultant expected distance statistics for each pair. This distance correlates with increasing sequence divergence between the respective pairs, as can be verified by their estimated $\mathrm{tim}e_{\text{marginal}}$ parameters and their corresponding expected fraction of changes to the amino acids at that inferred time. Note that the last 4 of 6 comparisons in the table yield negative compression ($\Delta_{\text{optimal}}<0$) for any single optimal alignment relationship inferred over those pairs. Nevertheless, the marginal model for the first five comparisons is able to assert that their respective sequences are indeed related ($\Delta_{\text{marginal}}>0$). Only for the last comparison between human hemoglobin with red-alga phycocyanin, the marginal loses to the null ($\Delta_{\text{marginal}}>0$), by a small amount (0.6 bits).

**Fig. 4.**
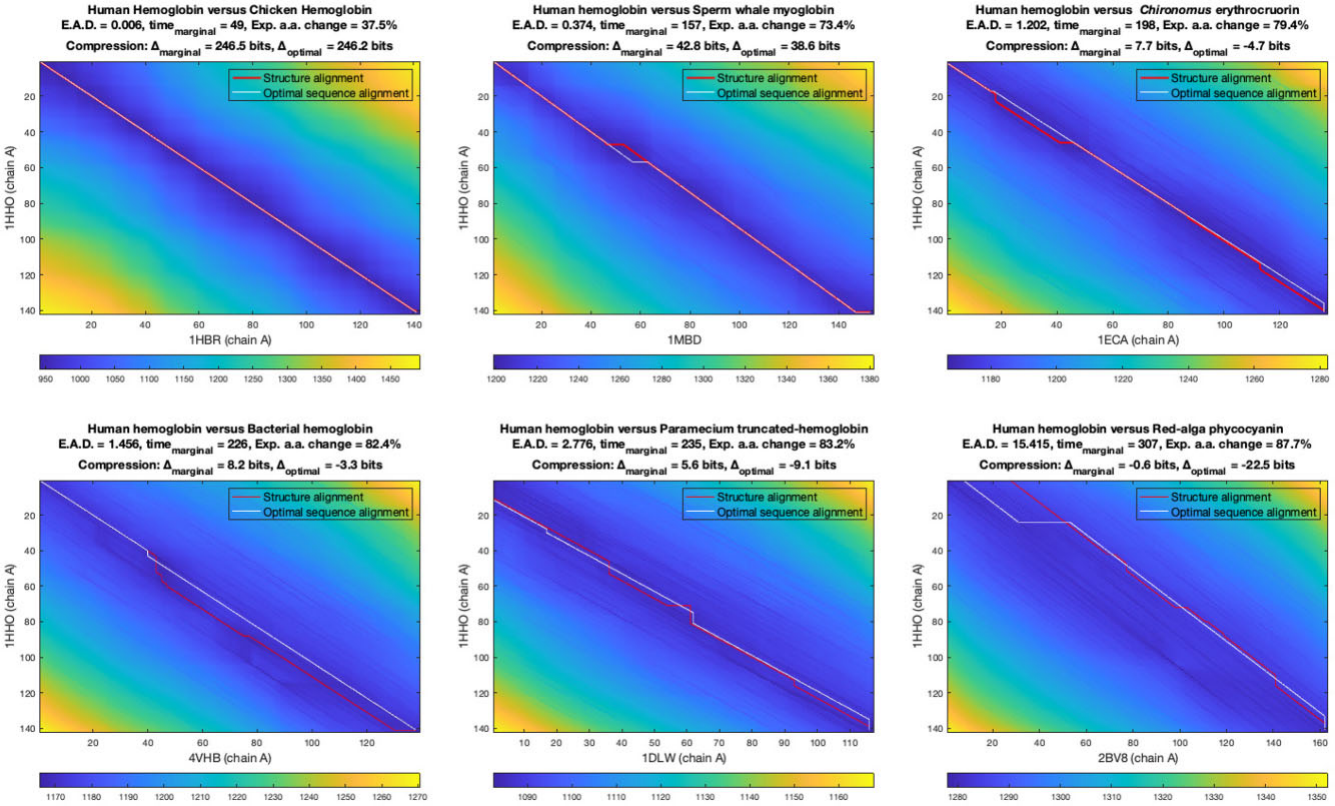
Visualization of the marginal probability matrices/landscapes generated after comparing the amino acid sequence of human hemoglobin (1HHO chain A) with the sequences of six other homologous globins. The color-codes denote the negative logarithm of the product of marginal probabilities that the prefixes and the suffixes are related (see Section 2.2). The colors within each matrix vary between the range of its [min,max] matrix values

Figure 4 gives the marginal probability landscapes where the fitness of all competing alignments can be visualized—as a color map—and the optimal sequence alignment and the corresponding reference structure alignment, appears as a source-to-sink path in white and red, respectively. The dark blue regions on the marginal landscape show the relative high-probability regions of sequence relationships. The expected distance measure presented here takes into account those and all other alignments (based on their posterior probabilities). The expected distance statistics for sequence comparisons in the daylight zone (e.g. human versus chicken hemoglobin) remains near 0 but, as the sequence pairs diverge in varying degrees into the twilight zone (all other comparisons, except the last), the expected distances increase as expected. The largest distance corresponds to the comparison of human hemoglobin with red-alga phycocyanin (expected distance of 15.4) where the sequences have diverged into the midnight zone of sequence relationship, with estimated $\mathrm{tim}e_{\text{marginal}}=307$ (or expected amino acid change = 87.7%). Previous studies have noted that phycocyanins and globins have an extremely distant evolutionary relationship with distinct ligands involved and biological functions associated with them, and are known to have no substantial sequence similarity (something confirmed by the statistics above) but with similar folding patterns (Pastore and Lesk, 1990).

In sum, we have performed a comprehensive analysis of the compatibility of alignments derived from sequences and structures of pairs of proteins. This analysis identifies the extent of sequence divergence between pairs beyond which the alignments agree closely with each other (the daylight zone of sequence relationship), the extent of sequence divergence where the alignments start to diverge and where any given residue-level alignment should be handled with caution (the twilight zone), and finally the extent of divergence beyond which it is impossible to detect any sequence relationship (the midnight zone) where any alignment generated from sequences alone should be discarded all together.

In contrast to previous attempts which deal with a single optimal sequence alignment computed using ad hoc choices for alignment parameters, the work presented here explicitly models the deteriorating quality of sequence relationships by employing marginal probabilities computed rigorously using time-parameterized statistical models. Another key difference of this work compared to the work of Rost (1999) is the latter’s reliance on a simple binary ‘yes’/‘no’ notion of structural similarity in estimating the three zones of sequence relationship. In comparison, this work rigorously computes the expected inter-alignment distance between sequences and structures at a residue level using strict probabilistic models and explores the limits of residue–residue relationships from sequence information alone.

As future work, it would be interesting to examine, using the developed framework of sequence comparison, probabilistic models of amino acid relationships and measures of inter-alignment distance, the specified relationships and classification in popular databases such as SCOP (Murzin *et al.*, 1995), CATH (Orengo *et al.*, 1997) and ECOD (Cheng *et al.*, 2014).

*Financial Support*: none declared.

*Conflict of Interest*: none declared. 

## Supplementary Material

btac247_Supplementary_Data
