# Supplementary material for: On the reliability and the limits of inference of amino acid sequence alignments
Source: Bioinformatics. 2022 Jun 27;38(Suppl 1):i255–63. doi: 10.1093/bioinformatics/btac247 (PMC9235515; doi:10.1093/bioinformatics/btac247)
Supplement: btac247_Supplementary_Data [file btac247_supplementary_data.pdf]

Supplementary Notes for:

On the reliability and the limit of inference of amino acid sequence alignments

Sandun Rajapaksa,[1] Dinithi Sumanaweera,[1,2] Arthur Lesk,[3] Lloyd Allison,[1] Peter J. Stuckey,[1] Maria Garcia de la Banda,[1] David Abramson,[4] and Arun S. Konagurthu [1,*]

[1]Department of Data Science and Artificial Intelligence, Faculty of Information Technology, Monash University, Clayton, VIC 3800, Australia
[2] Current address: Wellcome Sanger Institute , Wellcome Genome Campus, Cambridge, UK
Department of Data Science and Artificial Intelligence, Faculty of Information Technology, Monash University, Clayton, VIC 3800, Australia
[3]Department of Biochemistry and Molecular Biology and Center for Computational Biology and Bioinformatics, The Pennsylvania State University, University Park PA 16802, U.S.A.
[4]Research Computing Center, University of Queensland, St Lucia, QLD, Australia.

S1 Derivation of EAD recurrences given in the main text Section 3.2

Beyond the notations introduced in section 3.2 of the main text, the derivation below uses the following additional ones:

$\mathbf{A}$	the **set** of all possible alignments between $\langle S, T\rangle$.
$\mathbf{A}_{(i,j)}$	the **set** of all possible alignments of their prefixes $\langle S_{1\dots i}, T_{1\dots j}\rangle$ of the sequences.
$\mathbf{A}^{\mathtt{m}}_{(i,j)}$	the **subset** of all alignments of prefixes that end in a `match(m)` state at cell (i, j).
$\mathbf{A}^{\mathtt{i}}_{(i,j)}$	the **subset** of all alignments of prefixes that end in a `insert(i)` state at cell (i, j).
$\mathbf{A}^{\mathtt{d}}_{(i,j)}$	the **subset** of all alignments of prefixes that end in a `delete(d)` state at cell (i, j).
$\mathcal{A}^{\mathtt{m}}_{(i,j)}$	any **alignment** of prefixes that ends in a `match(m)` state at (i, j).
$\mathcal{A}^{\mathtt{i}}_{(i,j)}$	any **alignment** of prefixes that ends in a `insert(i)` state at (i, j).
$\mathcal{A}^{\mathtt{d}}_{(i,j)}$	any **alignment** of prefixes that ends in a `delete(d)` state at (i, j).
$\mathcal{A}^{\mathtt{m}\vert\mathtt{m}}_{(i,j)}$	any **alignment** of prefixes that ends in a `match(m)` state at (i, j) given a `match(m)` state at $(i-1, j-1)$. (Similar notation for all 9 possible transitions going between any two states of {`match`, `insert`, `delete`}.)
$\Pr(\mathtt{m}\vert\mathtt{m})$	the transition probability of going into a `match` given a previous `match` state. (Similar notation for all 9 possible transitions going between any two states of {`match`, `insert`, `delete`}.)
$\Pr(\langle s_i, t_j\rangle)$	the joint probability of matching a pair of amino acids, $s_i \in S$ and $t_j \in T$.

Derivation

Starting with recurrence (6) in the main text, by the definition of $EAD^{\mathtt{m}}(i, j)$, we have:

$$EAD^{\mathtt{m}}(i,j) = \sum_{\forall \mathcal{A}^{\mathtt{m}}_{(i,j)} \in \mathbf{A}^{\mathtt{m}}_{(i,j)}} \Pr(\mathcal{A}^{\mathtt{m}}_{(i,j)}, \langle S_{1\dots i}, T_{1\dots j}\rangle) \times \text{distance}(\mathcal{A}^{\mathtt{m}}_{(i,j)}, \mathcal{A}_{\text{ref}}), \quad (1)$$

But all alignments $\mathbf{A}^{\mathtt{m}}_{(i,j)}$ that end in a `match (m)` state at (i, j) are derived by extending all alignments arriving at the cell $(i-1, j-1)$ in any of the three alignment states ({`match`, `insert`, `delete`}), that is the set of alignments $\mathbf{A}_{(i-1,j-1)} = \mathbf{A}^{\mathtt{m}}_{(i-1,j-1)} \cup \mathbf{A}^{\mathtt{i}}_{(i-1,j-1)} \cup \mathbf{A}^{\mathtt{d}}_{(i-1,j-1)}$, by a pair of matched amino acids corresponding to the cell (i, j), that is, $\langle s_i, t_j\rangle$.

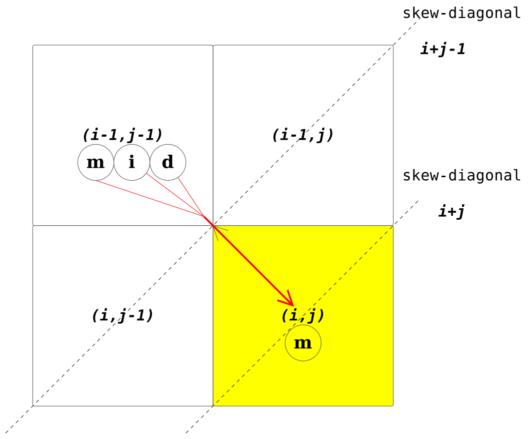

Figure SF1. All alignments ending in a `match` state at (i, j) cross two skew-diagonals, along which their additional distances has to be accounted for during dynamic programming

Therefore, Equation 1, can be decomposed based on the above observation as:

$$
\begin{aligned}
EAD^{\mathtt{m}}(i,j) \;=\; & \sum_{\forall \mathcal{A}^{\mathtt{m|m}}_{(i,j)} \in \mathbf{A}^{\mathtt{m}}_{(i,j)}} \Pr(\mathcal{A}^{\mathtt{m|m}}_{(i,j)}, \langle S_{1\dots i}, T_{1\dots j}\rangle) \times \text{distance}(\mathcal{A}^{\mathtt{m|m}}_{(i,j)}, \mathcal{A}_{\text{ref}}) \\
+ & \sum_{\forall \mathcal{A}^{\mathtt{m|i}}_{(i,j)} \in \mathbf{A}^{\mathtt{m}}_{(i,j)}} \Pr(\mathcal{A}^{\mathtt{m|i}}_{(i,j)}, \langle S_{1\dots i}, T_{1\dots j}\rangle) \times \text{distance}(\mathcal{A}^{\mathtt{m|i}}_{(i,j)}, \mathcal{A}_{\text{ref}}) \\
+ & \sum_{\forall \mathcal{A}^{\mathtt{m|d}}_{(i,j)} \in \mathbf{A}^{\mathtt{m}}_{(i,j)}} \Pr(\mathcal{A}^{\mathtt{m|d}}_{(i,j)}, \langle S_{1\dots i}, T_{1\dots j}\rangle) \times \text{distance}(\mathcal{A}^{\mathtt{m|d}}_{(i,j)}, \mathcal{A}_{\text{ref}})
\end{aligned} \tag{2}
$$

where the component joint probability terms in the r.h.s of Equation 2 are equivalent to:

$$
\begin{aligned}
\Pr(\mathcal{A}^{\mathtt{m|m}}_{(i,j)}, \langle S_{1\dots i}, T_{1\dots j}\rangle) &= \Pr(\mathcal{A}^{\mathtt{m}}_{(i-1,j-1)}, \langle S_{1\dots i-1}, T_{1\dots j-1}\rangle) \times \Pr(\mathtt{m|m}) \times \Pr(\langle s_i, t_j\rangle) \\
\Pr(\mathcal{A}^{\mathtt{m|i}}_{(i,j)}, \langle S_{1\dots i}, T_{1\dots j}\rangle) &= \Pr(\mathcal{A}^{\mathtt{i}}_{(i-1,j-1)}, \langle S_{1\dots i-1}, T_{1\dots j-1}\rangle) \times \Pr(\mathtt{m|i}) \times \Pr(\langle s_i, t_j\rangle) \\
\Pr(\mathcal{A}^{\mathtt{m|d}}_{(i,j)}, \langle S_{1\dots i}, T_{1\dots j}\rangle) &= \Pr(\mathcal{A}^{\mathtt{d}}_{(i-1,j-1)}, \langle S_{1\dots i-1}, T_{1\dots j-1}\rangle) \times \Pr(\mathtt{m|d}) \times \Pr(\langle s_i, t_j\rangle)
\end{aligned}
$$

Further, the component distance terms in the r.h.s of Equation 2 can be expanded as:

$$
\begin{aligned}
\text{distance}(\mathcal{A}^{\mathtt{m|m}}_{(i,j)}, \mathcal{A}_{\text{ref}}) &= \text{distance}(\mathcal{A}^{\mathtt{m}}_{(i-1,j-1)}, \mathcal{A}_{\text{ref}}) + \delta(i+j-1) + \delta(i+j) \\
\text{distance}(\mathcal{A}^{\mathtt{m|i}}_{(i,j)}, \mathcal{A}_{\text{ref}}) &= \text{distance}(\mathcal{A}^{\mathtt{i}}_{(i-1,j-1)}, \mathcal{A}_{\text{ref}}) + \delta(i+j-1) + \delta(i+j) \\
\text{distance}(\mathcal{A}^{\mathtt{m|d}}_{(i,j)}, \mathcal{A}_{\text{ref}}) &= \text{distance}(\mathcal{A}^{\mathtt{d}}_{(i-1,j-1)}, \mathcal{A}_{\text{ref}}) + \delta(i+j-1) + \delta(i+j)
\end{aligned}
$$

This holds because any alignment ending in a `match` state at (i, j) must arrive from $(i-1, j-1)$, and in doing so, will cross two skew-diagonals (see Figure SF1. Also cf. Figure 1 in the main text)

1. one skew-diagonal indexed by $i+j-1$ and
2. the other skew-diagonal indexed by $i+j$.

Thus, for all alignments going from $(i-1, j-1)$ to (i, j), the component distance terms at $(i-1, j-1)$ get augmented by a $\delta(i+j-1)+\delta(i+j)$, accounting for their widths/slacks with respect to the reference alignment $\mathcal{A}_{\text{ref}}$ along the above two skew-diagonals.

Substituting the expanding component joint probability and distance terms shown above into Equation 2, after rearranging yields:

$$\begin{aligned}
EAD^{\mathtt{m}}(i,j) = & \underbrace{\sum_{\forall \mathcal{A}^{\mathtt{m}}_{(i-1,j-1)} \in \mathbf{A}^{\mathtt{m}}_{(i-1,j-1)}} \Pr(\mathcal{A}^{\mathtt{m}}_{(i-1,j-1)}, \langle S_{1\ldots i-1}, T_{1\ldots j-1}\rangle) \times \mathrm{distance}(\mathcal{A}^{\mathtt{m}}_{(i-1,j-1)}, \mathcal{A}_{\mathrm{ref}})}_{EAD^{\mathtt{m}}(i-1,j-1)} \times \Pr(\mathtt{m}|\mathtt{m}) \times \Pr(\langle s_i, t_j\rangle) \\
& + \sum_{\forall \mathcal{A}^{\mathtt{m}}_{(i-1,j-1)} \in \mathbf{A}^{\mathtt{m}}_{(i-1,j-1)}} \Pr(\mathcal{A}^{\mathtt{m}}_{(i-1,j-1)}, \langle S_{1\ldots i-1}, T_{1\ldots j-1}\rangle) \times \Pr(\mathtt{m}|\mathtt{m}) \times \Pr(\langle s_i, t_j\rangle) \times [\delta(i+j-1) + \delta(i+j)] \\
+ & \underbrace{\sum_{\forall \mathcal{A}^{\mathtt{i}}_{(i-1,j-1)} \in \mathbf{A}^{\mathtt{i}}_{(i-1,j-1)}} \Pr(\mathcal{A}^{\mathtt{i}}_{(i-1,j-1)}, \langle S_{1\ldots i-1}, T_{1\ldots j-1}\rangle) \times \mathrm{distance}(\mathcal{A}^{\mathtt{i}}_{(i-1,j-1)}, \mathcal{A}_{\mathrm{ref}})}_{EAD^{\mathtt{i}}(i-1,j-1)} \times \Pr(\mathtt{m}|\mathtt{i}) \times \Pr(\langle s_i, t_j\rangle) \\
& + \sum_{\forall \mathcal{A}^{\mathtt{i}}_{(i-1,j-1)} \in \mathbf{A}^{\mathtt{i}}_{(i-1,j-1)}} \Pr(\mathcal{A}^{\mathtt{i}}_{(i-1,j-1)}, \langle S_{1\ldots i-1}, T_{1\ldots j-1}\rangle) \times \Pr(\mathtt{m}|\mathtt{i}) \times \Pr(\langle s_i, t_j\rangle) \times [\delta(i+j-1) + \delta(i+j)] \\
+ & \underbrace{\sum_{\forall \mathcal{A}^{\mathtt{d}}_{(i-1,j-1)} \in \mathbf{A}^{\mathtt{d}}_{(i-1,j-1)}} \Pr(\mathcal{A}^{\mathtt{d}}_{(i-1,j-1)}, \langle S_{1\ldots i-1}, T_{1\ldots j-1}\rangle) \times \mathrm{distance}(\mathcal{A}^{\mathtt{d}}_{(i-1,j-1)}, \mathcal{A}_{\mathrm{ref}})}_{EAD^{\mathtt{d}}(i-1,j-1)} \times \Pr(\mathtt{m}|\mathtt{d}) \times \Pr(\langle s_i, t_j\rangle) \\
& + \sum_{\forall \mathcal{A}^{\mathtt{d}}_{(i-1,j-1)} \in \mathbf{A}^{\mathtt{d}}_{(i-1,j-1)}} \Pr(\mathcal{A}^{\mathtt{d}}_{(i-1,j-1)}, \langle S_{1\ldots i-1}, T_{1\ldots j-1}\rangle) \times \Pr(\mathtt{m}|\mathtt{d}) \times \Pr(\langle s_i, t_j\rangle) \times [\delta(i+j-1) + \delta(i+j)]
\end{aligned} \tag{3}$$

By grouping all even terms on the r.h.s. of Equation 3 together, we get the recurrence:

$$\begin{aligned}
EAD^{\mathtt{m}}(i,j) = \; & EAD^{\mathtt{m}}(i-1,j-1) \times \Pr(\mathtt{m}|\mathtt{m}) \times \Pr(\langle s_i, t_j\rangle) \\
+ \; & EAD^{\mathtt{i}}(i-1,j-1) \times \Pr(\mathtt{m}|\mathtt{i}) \times \Pr(\langle s_i, t_j\rangle) \\
+ \; & EAD^{\mathtt{d}}(i-1,j-1) \times \Pr(\mathtt{m}|\mathtt{d}) \times \Pr(\langle s_i, t_j\rangle) \\
+ \; & \Bigg(\sum_{\forall \mathcal{A}^{\mathtt{m}}_{(i-1,j-1)} \in \mathbf{A}^{\mathtt{m}}_{(i-1,j-1)}} \Pr(\mathcal{A}^{\mathtt{m}}_{(i-1,j-1)}, \langle S_{1\ldots i-1}, T_{1\ldots j-1}\rangle) \times \Pr(\mathtt{m}|\mathtt{m}) \times \Pr(\langle s_i, t_j\rangle) \\
& + \sum_{\forall \mathcal{A}^{\mathtt{i}}_{(i-1,j-1)} \in \mathbf{A}^{\mathtt{i}}_{(i-1,j-1)}} \Pr(\mathcal{A}^{\mathtt{i}}_{(i-1,j-1)}, \langle S_{1\ldots i-1}, T_{1\ldots j-1}\rangle) \times \Pr(\mathtt{m}|\mathtt{i}) \times \Pr(\langle s_i, t_j\rangle) \\
& + \sum_{\forall \mathcal{A}^{\mathtt{d}}_{(i-1,j-1)} \in \mathbf{A}^{\mathtt{d}}_{(i-1,j-1)}} \Pr(\mathcal{A}^{\mathtt{d}}_{(i-1,j-1)}, \langle S_{1\ldots i-1}, T_{1\ldots j-1}\rangle) \times \Pr(\mathtt{m}|\mathtt{d}) \times \Pr(\langle s_i, t_j\rangle) \Bigg) \\
& \times [\delta(i+j-1) + \delta(i+j)]
\end{aligned} \tag{4}$$

But the last term on the r.h.s. is the marginal probability over all alignments ending in a `match` at (i, j), resulting in the final form of recurrence (6) used in the main text:

$$\begin{aligned}
EAD^{\mathtt{m}}(i,j) = \; & EAD^{\mathtt{m}}(i-1,j-1) \times \Pr(\mathtt{m}|\mathtt{m}) \times \Pr(\langle s_i, t_j\rangle) \\
+ \; & EAD^{\mathtt{i}}(i-1,j-1) \times \Pr(\mathtt{m}|\mathtt{i}) \times \Pr(\langle s_i, t_j\rangle) \\
+ \; & EAD^{\mathtt{d}}(i-1,j-1) \times \Pr(\mathtt{m}|\mathtt{d}) \times \Pr(\langle s_i, t_j\rangle) \\
+ \; & \Pr_{\text{marginal}}(\langle S_{1\ldots i}, T_{1\ldots j}\rangle \, |\mathtt{match@}(i,j)) \times [\delta(i+j-1) + \delta(i+j))
\end{aligned} \tag{5}$$

Recurrences (7) and (8) in the main text follow identical lines of derivations, with the only difference that they account for all alignments coming into (i, j) in a $\mathtt{insert}(i)$ and $\mathtt{delete}(d)$ states, respectively. Also, all such alignment transitions only cross a single skew-diagonal, indexed by $i + j$, therefore those recurrences will contain only the $\delta(i + j)$ term.

S2 Choice of the data source

S2.1 Set of million domain pairs

In this work we randomly sampled a million domain pairs from the Structural Classification of Proteins (SCOPe v2.07) database [Murzin et al., 1995] using the random sampling method described below. The full list of scop domain pairs (including the SCOP domain identifiers and the SCOP classification level) can be downloaded from here.

S2.1.1 Random sampling method

A domain pair is randomly selected by utilising the SCOP organisation of domains within its hierarchical classification tree. The internal nodes of this tree are associated with the four-level classification of protein domains: `class`, `fold`, `superfamily` and `family`. Each domain is uniquely represented by a leaf node. A traversal from the root node to a leaf node yields a domain. The sampling procedure involves traversing from the root to a leaf node, passing each of the SCOP levels: `class`, `fold`, `superfamily` and `family` in order. At each node of this traversal, until a leaf node (domain) is reached, a child node is selected from the available children (i.e. nodes in the level below the current node), by sampling randomly based on the weights (i.e. number of leaves) of their respective subtrees. Thus, to identify domain pairs within the same `superfamily` but under different families, the traversal first proceeds from the root to the `superfamily` level. Then the weighted random sampling method selects two random domains (leaves) from two different families (child nodes). To identify pairs from the same `family`, a pair of its children (leaves) are randomly selected, when the traversal reaches `family` level nodes, while considering families with more than 2 domains.

S2.2 Five sets of domain pairs sampled at varying levels of SCOP hierarchy

Using the random sampling method described above, we further sampled sets of unique domain pairs from each hierarchical level of SCOP such that each domain appears at most once in the dataset. This comprised 5 sets of domain pairs sampled at the same `family`, same `superfamily`, same `fold`, same `class`, and `decoy` (different class) levels respectively. See Table ST1 for more information on the datasets.

Table ST1. Statistics of the sampled sets of domain pairs at each SCOP level

SCOP Level	Number of unique domain pairs sampled	Number of unique domains in the set
Family	55,121	110,242
SuperFamily	31,585	63,170
Fold	40,567	81,134
Class	40,517	81,034
Decoy	40,433	80,866

S3 Results for the set of million domain pairs

S3.1 Variation of the expected inter-alignment distance as a function of the Markov time parameter

We computed the expected inter-alignment distance and the inferred Markov time parameter ($\texttt{time}_{\text{marginal}}$) for each of the million domain pairs, over all possible combinations of the 4 time-parameterized models: BLOSUM [Henikoff and Henikoff, 1992], PAM [Dayhoff et al., 1978], MML-SUM [Sumanaweera et al., 2020] and VTML [Müller et al., 2002] and the 3 reference structure alignment programs: `TM-align` [Zhang and Skolnick, 2005], `MMLigner` [Collier et al., 2017] and `DALI` [Holm and Sander, 1995].

Each of these 12 possible combinations generated a million data points respectively. For each comparison, these million data points were grouped into bins in the range $[1, 500]$ based on the inferred Markov time ($\texttt{time}_{\text{marginal}}$) (bottom x-axes of the plots in Fig. SF2) . Then, for each bin the first (Q1), second (Q2) and third (Q3) quartile statistics of the excepted inter-alignment distances were computed. Fig. SF2 shows the variation of the expected inter-alignmet distance versus $\texttt{time}_{\text{marginal}}$ for all the 12 possible combinations. The brown curve in the Fig. SF2 shows the % cumulative growth whose vertical scale appears on the right side of each plot. The top x-axes of the plots shows the expected %-change of amino acids in the range of $[1\%, \sim 92\%]$ which corresponds to the time-range $[1, 500]$ for each time-parameterized models which is available here (see Sumanaweera et al. [2019] for more details).

S3.1.1 Canonicalization of reference alignments

For the computation of expected inter-alignment distance, the insert and delete blocks of all the reference (structure) alignments produced by the 3 reference alignment programs (`MMLigner`, `TM-Align`, and `DALI`) were converted to $\texttt{D}^*\texttt{I}^*$ form as shown in the example below.

Consider an alignment between two sequences S and T:

```
S     KRVGKRLNIQLKKGTEGLGFSITSRDVTIGGSAPI-Y-V--KNILPRGAAIQDGRLKAGD
T     --------IKRGLLGIKGTEMSADIAKAFNLDVQRG-F-SE--VLPGSGSAKAG-VKAGD

S    RLIEVNGV--AGKSQEEVVSLLRSTKMEGTVSLLVFRQEEA----------
T    IITSLNGKPL--NSFAELRSRIATTEPGTKVKLGLLRNGKPLEVEVTLDTS
```

After canonicalizing the alignment in the form $\texttt{D}^*\texttt{I}^*$:

```
S     KRVGKRLNIQLKKGTEGLGFSITSRDVTIGGSAPIYVKN----ILPRGAAIQDGRLKAGD
```

```
T   --------IKRGLLGIKGTEMSADIAKAFNLDVQR----GFSEVLPGSGSAKAG-VKAGD

S   RLIEVNGVAG--KSQEEVVSLLRSTKMEGTVSLLVFRQEEA----------
T    IITSLNGK--PLNSFAELRSRIATTEPGTKVKLGLLRNGKPLEVEVTLDTS
```

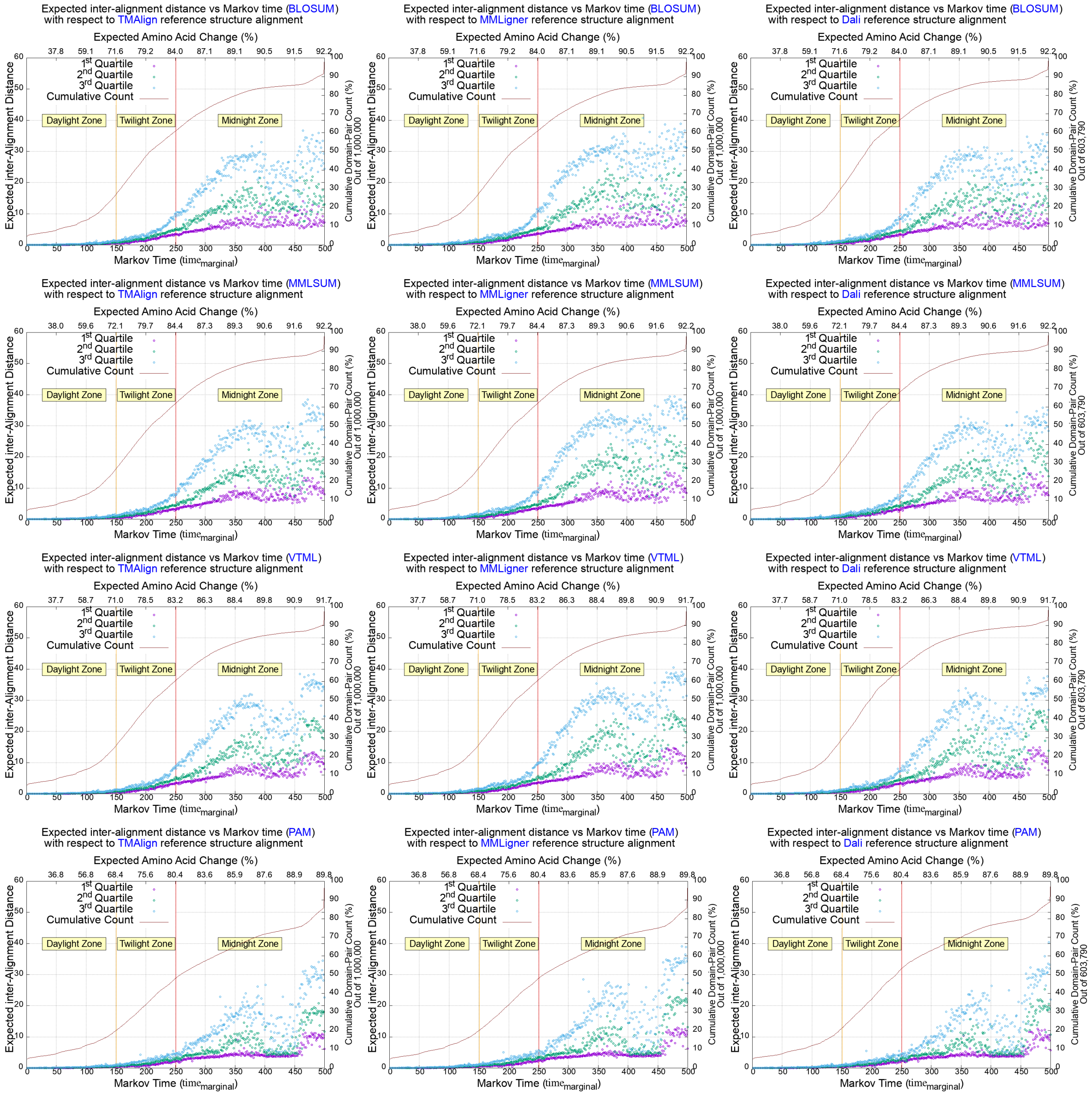

Figure SF2. First, second and third quartile statistics of the expected inter-alignment distance with respect to a given reference alignment as a function of the inferred Markov time parameter, $\texttt{time}_{\text{marginal}}$. Each row corresponds to the 4 time-parameterized models employed during the MML sequence comparision: BLOSUM, MMLSUM, VTML and PAM in order. Each column from left to right corresponds to the statistics obtained using as reference the structure alignment from `TM-Align`, `MMLigner` and `DALI` respectively. In each plot the x-axis (shown below the box) is the Markov time step in the range $[1, 500]$; the range on the top of the plot is the corresponding expected %-change of amino acids for the chosen time-parameterized model; the y-axis (left of the box) is the expected distance; the scatter plots (magenta, green, and blue) track the changes in the 1st, 2nd, and 3rd quartile statistic of the expected distance statistic over 1 million domain pairs, grouped according to their inferred integer Markov time step ($\texttt{time}_{\text{marginal}}$); the vertical range on the right of the box tracks the cumulative %-growth of the number of domain pairs as a function of time on the x-axis.

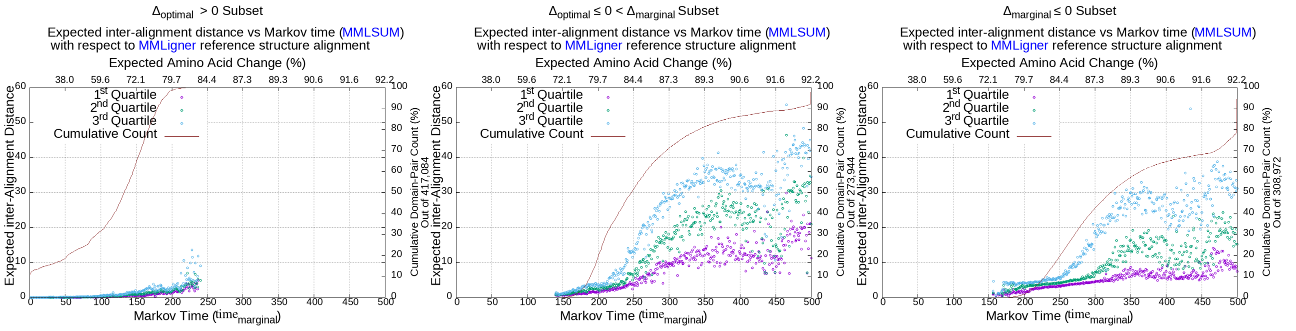

(a) (b) (c)

Figure SF3. Expected inter-alignment distance as a function of the inferred Markov time parameter using MMLSUM model with respect to `MMLigner` reference structure alignments after separating the million domain-pairs into distinct groups based on their compression statistics

Note that the `DALI` structure alignment program resulted only 603,790 valid alignments out of the million domain pairs. It failed to produce an alignment relationship for some domain pairs containing multiple chains and for some domain pairs, it changed the amino acid residues in either or both of the sequences reported in the alignment corresponding to the PDB files. Therefore, these alignments were ignored in the analysis.

S3.2 Variation of the expected inter-alignment distance as a function of Markov time parameter after separating the million domain-pairs into distinct groups based on their compression statistics

We grouped the million domain pairs into three subsets based on the respective statistical significance test statistics (Δ_{optimal} and Δ_{marginal}).

1. The first group accounts for the $417,084$ domain pairs whose optimal sequence alignment under the MML framework yielded positive compression (i.e., $\Delta_{\text{optimal}} > 0$).
2. The second group accounts for $273,944$ domain pairs whose optimal sequence alignment lost to the null model but gave positive compression using the marginal model (i.e., $\Delta_{\text{optimal}} \leq 0 < \Delta_{\text{marginal}}$).
3. The final group contains the remaining $308,972$ domain pairs where $\Delta_{\text{marginal}} \leq 0$.

Figure SF3 shows the variation of the expected inter-alignment distance statistic as a function of the inferred Markov time parameter using MMLSUM time-parameterized model with respect to the reference structure alignments reported by `MMLigner` for the above distinct groups of domain pairs. (see supplementary website for the remaining combinations.)

S3.3 Variation of the empirical inter-alignment distance as a function of Markov time parameter

S3.3.1 Random sampling approach used to generate highly-probable alignments

For any two sequences S and T an alignment can be sampled as a three-state string of **`match`**, **`insert`**, **`delete`** states based on a *Roulette wheel selection*. Here the objective is to give every state a chance of being selected, but to make more probable states at a certain position in the alignment string more likely to be chosen over the other states. This is achieved by allocating a section in an imaginary roulette wheel where the sections are of different sizes, proportional to the marginal probability of the individual states.

Running `seqMMLigner` in its `marginal` mode generates three matrices (one for each state) containing marginal probabilities of the two sequences [Sumanaweera et al., 2019]. Given the marginal probability matrices of the two sequences S and T: $Tot_{\mathtt{m}}$, $Tot_{\mathtt{i}}$, and $Tot_{\mathtt{d}}$, we create an imaginary roulette wheel for the $(i, j)^{\text{th}}$ position of the alignment string. Here, the size of the section of each state is defined using the marginal probability of the three states at the given position, i.e., $Tot_{\mathtt{m}}(i, j)$, $Tot_{\mathtt{i}}(i, j)$ and $Tot_{\mathtt{d}}(i, j)$. Then we spin the wheel and select the state associated with the winning section. If the selected state is **`m`**, a new imaginary roulette wheel is created and spun to select the next state in the sampling method (previous state in the alignment) at $(i-1, j-1)^{\text{th}}$ position. If the **`i`** state is selected, the next roulette wheel selection is done on the $(i-1, j)^{\text{th}}$ position. Similarly, for the **`d`** state, the next roulette wheel selection is done at the position $(i, j-1)$. Starting from the $(|S|, |T|)^{\text{th}}$ position, this process is continued until the $(0, 0)^{\text{th}}$ position to generate a three-state string.

We continued this process multiple times to generate 1,000 randomly sampled three-state strings to cross-verify the computation of the mathematical expectation of distance, using an empirical (sampling) approach for million domain pairs. Then, the distance between each sampled alignment for a pair was compared to its corresponding reference structure alignment. The resulting estimates of distance were then averaged to generate an *empirical* estimate of the distance that can be compared against the computation of the mathematical expectation of inter-alignment distance. Fig. SF5 shows the variation of the empirical inter-alignment distance as a function of the inferred Markov time parameter, $\mathtt{time}_{\text{marginal}}$ for the 12 combinations discussed previously.

S3.4 Comparsion of Expected inter-alignment distance and E-value

Consider two random sequences with lengths m and n respectively. Given the distribution of individual amino acid residues, and a scoring matrix, the number of distinct local alignments with a score S is said to be Poisson distributed with a mean statistic of:

$$E(S) \approx K \times m \times n \times \exp(-\lambda \times S) \tag{6}$$

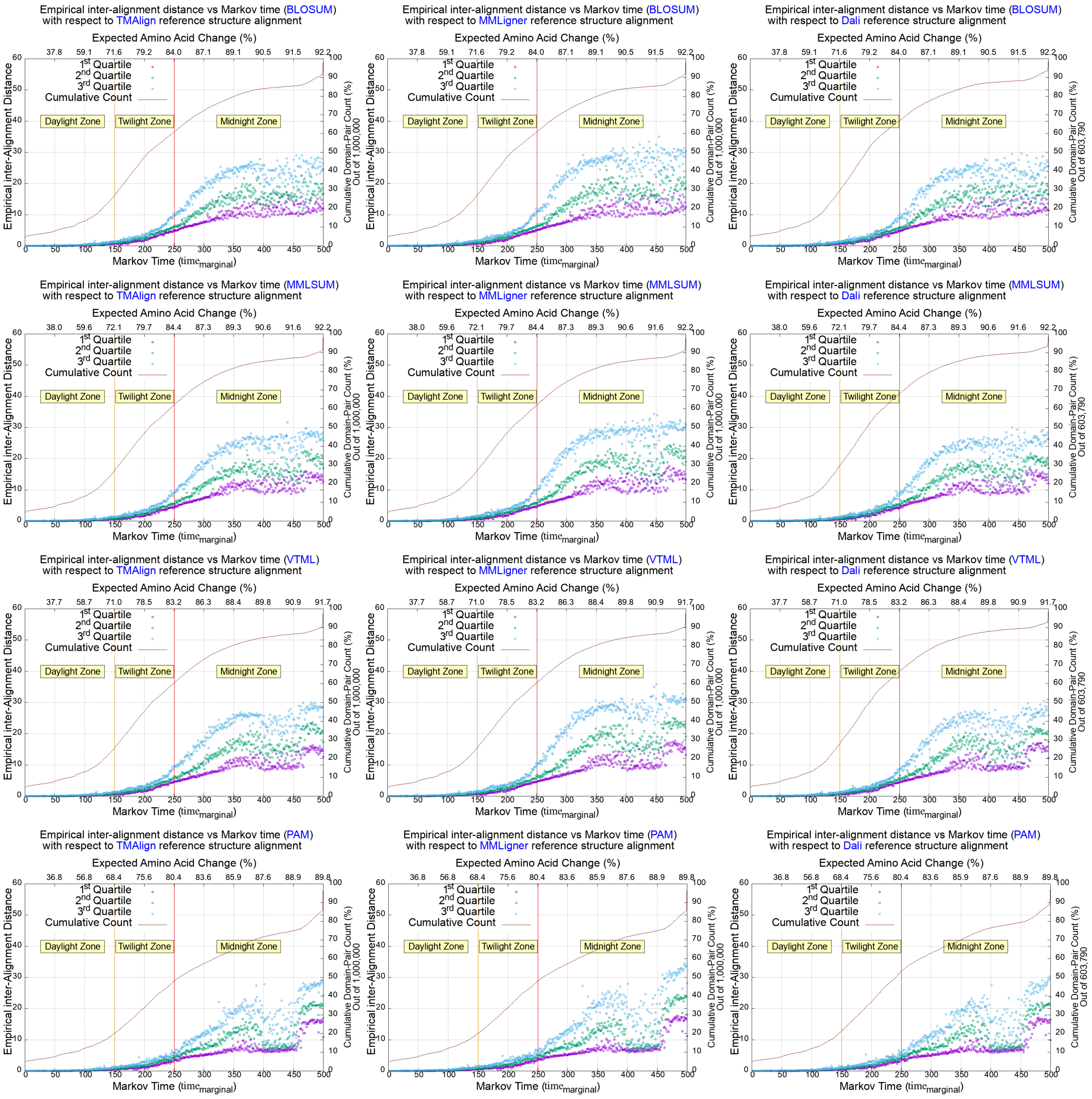

Figure SF4. First, second and third quartile statistics of the empirical inter-alignment distance with respect to a given reference alignment as a function of the inferred Markov time parameter, $\texttt{time}_{\text{marginal}}$. The legends of the plots mirrors that of Figure SF2

where λ and K can be calculated from the scoring matrix and the average sequence compositions based on the Poisson distribution. This is known as the E-value which gives the expected number of distinct optimal local alignments with a score of at least S [Karlin and Altschul, 1990, Altschul et al., 2001].

A subsequent study by Levitt and Gerstein [1998] have compared sequence alignment significance against structure alignment significance using the resultant E-values, and identified a statistical significance threshold of 1% which agrees for both types of alignments. With this work, they substantiated E-value as a common ground to evaluate alignment scores across different programs.

Here we computed the E-value for the million structure alignments generated by `TM-Align` program using the BLOSUM62 matrix and the associated default parameters used in Blast. Figure SF6 shows the variation of the E-value as a function of the inferred Markov time parameter ($\texttt{time}_{\text{marginal}}$) for the million domain pairs. Note that the left y-axis is in natural logarithmic scale.

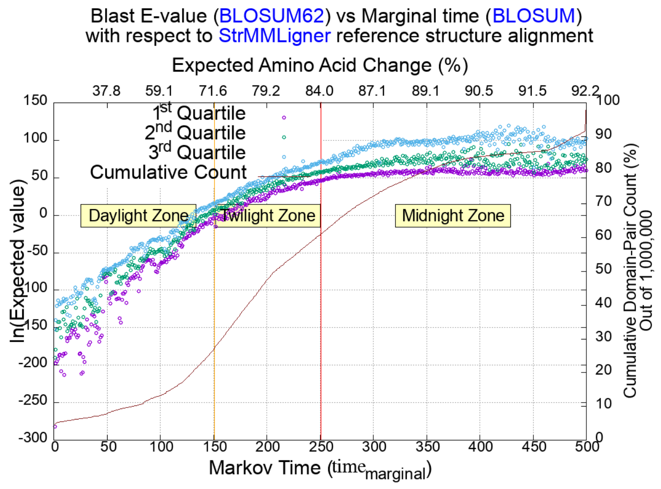

Figure SF5. First, second and third quartile statistics of the natural logarithm of E-value with respect to a reference alignment obtained using `TM-Align` program as a function of the inferred Markov time parameter using BLOSUM model.

In Figure SF6, the median of E-value falls below 0.01 in the time range $[1 - \sim 140]$ which corresponds to a expected % change $< \sim 70\%$. This is in agreement with the common wisdom where an E-value < 0.01 is considered a good hit for homology matches. [1]

S4 Results for the sets of domain pairs sampled from the 5 levels of SCOP

S4.1 Statistics of the 5 domain pairs sets

We randomly sampled sets of unique domain pairs from each hierarchical level of SCOP such that each domain appears at most once in the dataset. This comprised 5 sets of domain pairs sampled at the same `family`, `superfamily`, `fold`, `class`, and `decoy` levels respectively. See Table ST1 for more information on the datasets.

Next we computed the expected inter-alignment distance and the inferred Markov time parameter ($\texttt{time}_{\text{marginal}}$) for each set of domain pairs over all possible combinations of the 3 time-parameterized models: BLOSUM [Henikoff and Henikoff, 1992], MMLSUM [Sumanaweera et al., 2020] and VTML [Müller et al., 2002] and the 2 reference structure alignment programs: TM-align [Zhang and Skolnick, 2005], and MMLigner [Collier et al., 2017]. Each of these 6 possible combinations generated sets of data points respectively. The same set of steps were carried out to compute the first (Q1), second (Q2) and third (Q3) quartile statistics of the excepted inter-alignment distances. Fig. SF7 shows the variation of the expected inter-alignmet distance versus $\texttt{time}_{\text{marginal}}$ for the MMLSUM time parameterized model with respect to reference structure alignments reported by `MMLigner`.

References

S. F. Altschul, R. Bundschuh, R. Olsen, and T. Hwa. The estimation of statistical parameters for local alignment score distributions. *Nucleic Acids Research*, 29(2):351–361, 2001.

J. H. Collier, L. Allison, A. M. Lesk, P. J. Stuckey, M. Garcia de la Banda, and A. S. Konagurthu. Statistical inference of protein structural alignments using information and compression. *Bioinformatics*, 33(7):1005–1013, 2017.

M. Dayhoff, R. Schwartz, and B. Orcutt. A model of evolutionary change in proteins. *Atlas of Protein Sequence and Structure*, 5:345–352, 1978.

S. Henikoff and J. G. Henikoff. Amino acid substitution matrices from protein blocks. *Proceedings of the National Academy of Sciences*, 89(22): 10915–10919, 1992.

L. Holm and C. Sander. Dali: a network tool for protein structure comparison. *Trends in Biochemical Sciences*, 20(11):478–480, 1995.

S. Karlin and S. F. Altschul. Methods for assessing the statistical significance of molecular sequence features by using general scoring schemes. *Proceedings of the National Academy of Sciences*, 87(6):2264–2268, 1990.

M. Levitt and M. Gerstein. A unified statistical framework for sequence comparison and structure comparison. *Proceedings of the National Academy of sciences*, 95(11):5913–5920, 1998.

T. Müller, R. Spang, and M. Vingron. Estimating amino acid substitution models: a comparison of dayhoff's estimator, the resolvent approach and a maximum likelihood method. *Molecular Biology and Evolution*, 19(1):8–13, 2002.

A. G. Murzin, S. E. Brenner, T. Hubbard, and C. Chothia. SCOP: a structural classification of proteins database for the investigation of sequences and structures. *Journal of Molecular Biology*, 247(4):536–540, 1995.

[1] Details on these statistics are given by The National Center for Biotechnology Information at https://www.ncbi.nlm.nih.gov/BLAST/tutorial/Altschul-1.html

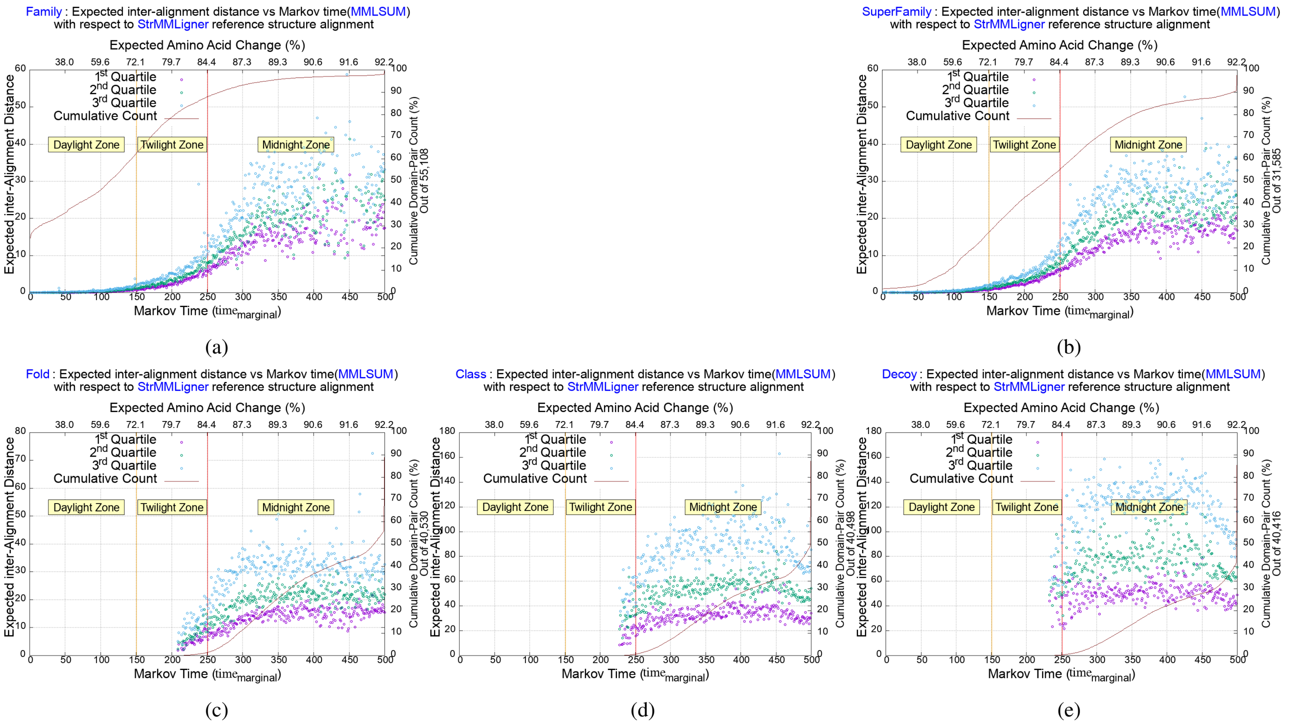

Figure SF6. The quartile statistics of the expected distance of sequence alignments with respect to a reference alignment obtained using `MMLigner` structure alignment program as a function of the inferred Markov time parameter, $\texttt{time}_{\text{marginal}}$ using MMLSUM model.

D. Sumanaweera, L. Allison, and A. S. Konagurthu. Statistical compression of protein sequences and inference of marginal probability landscapes over competing alignments using finite state models and dirichlet priors. *Bioinformatics*, 35(14):i360–i369, 2019.

D. Sumanaweera, L. Allison, and A. S. Konagurthu. Bridging the gaps in statistical models of protein alignment. *arXiv preprint arXiv:2010.00855*, 2020.

Y. Zhang and J. Skolnick. TM-align: a protein structure alignment algorithm based on the TM-score. *Nucleic Acids Research*, 33(7):2302–2309, 01 2005.
